# Nervensystem und Kommunikation: Polyneuropathie und die Rolle des peripheren Nervensystems

**DOI:** 10.1007/s00391-026-02593-y

**Published:** 2026-05-18

**Authors:** Bernhard Iglseder

**Affiliations:** Univ.-Klinik für Geriatrie der PMU, Uniklinikum Salzburg – Christian-Doppler-Klinik, Ignaz-Harrer-Str. 79, 5020 Salzburg, Österreich

**Keywords:** Altern, Lebensqualität, Autonomie, Abschwächung von Muskeleigenreflexen, Geriatrischer Ansatz, Aging, Quality of life, Autonomy, Weakening of muscle reflexes, Geriatric approach

## Abstract

Das zentrale Nervensystem (ZNS) verarbeitet Informationen, koordiniert Bewegungen und ermöglicht Kommunikation – sowohl innerhalb des Körpers als auch mit der Umgebung: Wahrnehmen und Reagieren auf Sinneseindrücke unterliegen der Steuerung des Gehirns, afferente und efferente Bahnen durchlaufen das Rückenmark. Das periphere Nervensystem (PNS) vermittelt zwischen Umgebung und ZNS. Mit zunehmendem Alter verändert sich das PNS, was zu einer Abschwächung von Muskeleigenreflexen und Propriozeption führt. Davon abzugrenzen sind Polyneuropathien (PNP) als Erkrankungen des PNS. Die Ursachen sind vielfältig: Neben isolierten metabolischen, immunmediierten, hereditären, toxischen und infektiösen Ätiologien können PNP auch Ausdruck von Systemerkrankungen sein. Neuropathien in Zusammenhang mit Diabetes, monoklonalen Gammopathien und Malignomen sind im Alter häufiger, auch der Anteil der kryptogenen Neuropathie nimmt im Alter zu. PNP tragen zu einer Beeinträchtigung der Mobilität und erhöhtem Sturzrisiko bei, was die Abklärung funktioneller Fähigkeiten erforderlich macht. Schmerzen und sensorische Störungen wirken sich negativ auf die Aktivitäten des täglichen Lebens aus. Als Folge wird die Kommunikation mit den Lebensumwelten beeinträchtigt. Ein geriatrischer Ansatz ermöglicht neben einer exakten Diagnose auch eine umfassende, auf die individuellen Bedürfnisse zugeschnittene Therapie.

## Abstract (en)

The central nervous system (CNS) processes information, coordinates movements, and enables communication—both within the body and with the environment: Perception and response to sensory impressions are controlled by the brain, with afferent and efferent pathways passing through the spinal cord. The peripheral nervous system (PNS) mediates between the environment and the CNS. As we age, the PNS undergoes changes, resulting in a weakening of muscle reflexes and proprioception. This must be distinguished from polyneuropathies (PNP), which are pathological changes in the PNS. The causes are manifold: in addition to metabolic, immune-mediated, hereditary, toxic, and infectious etiologies, polyneuropathies can be a manifestation of systemic diseases. Neuropathies associated with diabetes, monoclonal gammopathies, and malignancies are more common in older people, and the proportion of cryptogenic neuropathies also increases with age. PNPs contribute to impaired mobility and an increased risk of falls, which necessitates the assessment of functional abilities. Pain and sensory disturbances have a negative impact on activities of daily living. As a result, communication with the living environment is impaired. A geriatric approach enables not only an accurate diagnosis but also comprehensive therapy tailored to individual needs.

## Aufgaben und altersassoziierte Veränderungen des peripheren Nervensystems

Das periphere Nervensystem (PNS) umfasst alle Nerven außerhalb des zentralen Nervensystems (ZNS), das von Gehirn und Rückenmark gebildet wird. Das PNS besteht aus Hirn- und Spinalnerven, Ganglien sowie afferenten und efferenten Nervenfasern, die somatische und vegetative Funktionen steuern. Sensorische Nerven leiten Informationen, die man durch Sehen, Hören, Schmecken, Fühlen und Tasten aufnimmt, zum ZNS. Motorische Nerven leiten Befehlssignale vom ZNS zu den Muskeln (somatisches oder willkürliches PNS). Das vegetative (autonome) Nervensystem ist der Teil des PNS, der die inneren Organe (Blutgefäße, Magen, Darm, Leber, Nieren, Harnblase, Genitalien, Lunge, Pupillen, Herz, Schweiß- und Speichelfluss, Verdauungsdrüsen) versorgt. Die Kommunikation im Nervensystem erfolgt durch elektrochemische Signale. Das PNS spielt somit eine vielfältige Rolle bei Kommunikationsvorgängen, sowohl innerhalb von Nervensystem und Organismus als auch zwischen Individuum und Umwelt.

Das PNS ist im Laufe des Alternsprozesses Veränderungen unterworfen: An Einzelfaserpräparaten wurde bereits 1970 mit höherem Alter eine zunehmende Zahl abnormer Fasern mit Zeichen der Waller-Degeneration, segmentaler Demyelinisierung sowie Abnormitäten und Variationen der internodalen Distanz nachgewiesen. Die Zahl sympathischer Neurone im Rückenmark nimmt pro Dekade um etwa 8 % ab. Die Muskeleigenreflexe sind mit zunehmendem Alter an den unteren Extremitäten distal betont abgeschwächt, so ist der Achillessehnenreflex bei 38 % der > 65-Jährigen nicht auslösbar, die Reflexe der oberen Extremitäten fehlen lediglich bei 5 %. Das Berührungsempfinden zeigt mit dem Alter eine zunehmend höhere Schwelle, die Propriozeption leidet durch eine Reduktion der Mechanorezeptoren in Gelenken und Haut. Ein reduzierter Vibrationssinn, besonders distal an den Beinen, wurde bei 71 % einer gesunden Alterspopulation nachgewiesen [1]. Diese Alternsvorgänge des PNS führen in teils kausalem Zusammenspiel mit der Abnahme der Muskelmasse (Sarkopenie) zu einem Verlust von Muskelkraft und Gleichgewichtsfähigkeit. Daraus resultieren, zusätzlich beeinflusst von Alterungsphänomenen des ZNS, funktionelle Einschränkungen wie reduzierte Mobilität und erhöhtes Sturzrisiko [2, 3].

Von diesen physiologischen Altersveränderungen sind Polyneuropathien (PNP) abzugrenzen, die eine heterogene Gruppe von Erkrankungen des PNS darstellen. Das Auftreten von klinischen Symptomen (s. unten) und die Erhebung altersstandardisierter Werte in der elektrophysiologischen Untersuchung (NLG-Nervenleitgeschwindigkeit, EMG-Elektromyographie) erlauben die Abgrenzung von den genannten physiologischen Altersveränderungen. In der Rotterdam-Studie wurde bei älteren Probanden der Zusammenhang zwischen Alltagsfähigkeit, Stürzen und PNP untersucht: Bei Vorliegen einer PNP fand sich ein erhöhtes Sturzrisiko (OR 1,87, 95 %-KI 1,10–3,16), auch das Risiko, im Rahmen des Sturzes eine Fraktur zu erleiden, war hoch (OR 3,35, 95-KI 1,02–10,97). Die Betroffenen zeigten schlechtere Ergebnisse bei basalen und instrumentalen Aktivitäten des täglichen Lebens wie Gehvermögen, Mobilität oder Einkaufen. Auch Parameter der Ganganalyse (Gehgeschwindigkeit, Kadenz, Tandemgang) fielen bei Vorliegen einer PNP schlechter aus [4].

## Epidemiologie und Ätiologie im Alter

Die Inzidenz von PNP nimmt mit dem Alter zu: Von ca. 60/100.000 bei 50–54-Jährigen auf ca. 300/100.000 bei 75–79-Jährigen [5]. Eine Metaanalyse zeigt eine Prävalenz von 1 % für die Gesamtbevölkerung, die auf 3 % bei > 55-Jährigen und auf 7 % bei alten Menschen steigt [6]. Die Ursachen sind auch im Alter mannigfaltig: Neben metabolischen, immunmediierten, hereditären, toxischen und infektiösen Ätiologien treten PNP im Rahmen von Systemerkrankungen auf. Erwartungsgemäß nimmt im Alter die Inzidenz der diabetischen PNP zu, während z. B. immunmediierte Neuropathien abnehmen. Der Anteil an Neuropathien ohne eindeutige Ursache – kryptogene oder chronisch idiopathische Neuropathien – steigt mit dem Alter. Eine retrospektive Untersuchung an 785 Patienten > 65 Jahre fand eine kryptogene Neuropathie in 35,4 % und eine diabetische Neuropathie in 18,8 % (Tab. 1).

**Tab. 1:** Ursachen von Neuropathien bei 785 Patienten > 65 Jahre. (Nach Mathis et al. [7])

Ursache	Häufigkeit (%)
Idiopathisch	35,4
Diabetes	18,8
Immunologisch	7,9
Toxisch	7,4
Entzündlich	6
Hereditär	3,4
Infektiös	3
Alkohol	2,9
MGUS	2,9
Hämatologisch	2,5
Makroglobulinämie	2
Karzinom	2
Verschiedene metabolische Erkrankungen	2
Niereninsuffizienz	1,9
Primäre Amyloidose	1
Varia	1

*MGUS* monoklonale Gammopathie unklarer Signifikanz

Angesichts der im Alter häufigen Polypharmazie dürfen medikamenteninduzierte PNP nicht übersehen werden. Die Liste an PNP verursachenden Medikamenten ist lang, zu erwähnen sind vor allem Chemotherapeutika, einzelne Virostatika und Antibiotika sowie Statine und Amiodaron.

## Klinische Symptomatik

Klinische Zeichen von PNP umfassen motorische, sensible und autonome Störungen: Muskelkrämpfe, Faszikulationen oder Myokymien zählen zu den *positiven motorischen Symptomen* und deuten auf immunmediierte Neuropathien hin. Schwäche, Lähmungen und Muskelatrophie zählen zu den *negativen motorischen Symptomen, *die Ausdruck fortgeschrittener Stadien sind. *Positive sensible Symptome* wie Dysästhesien und Parästhesien helfen, erworbene von hereditären PNP zu unterscheiden, da bei Letzteren positive sensible Symptome selten sind. *Negative sensible Symptome* treten in Form von Hypästhesien auf. Informationen über Beginn und zeitlichen Verlauf erlauben eine weitere diagnostische Eingrenzung.

Autonome Symptome manifestieren sich als orthostatische Dysregulation, Obstipation oder Diarrhoe, Sexualfunktionsstörungen sowie Anhidrose. Im Alter findet sich eine autonome Beteiligung häufig bei den diabetischen und paraneoplastischen PNP. Ursächlich werden bei diesen Formen vor allem kleine, unmyelinisierte C‑Fasern und dünn myelinisierte Aδ-Fasern der autonomen Nerven geschädigt.

Die klinische Untersuchung muss differenzialdiagnostisch altersassoziierte Veränderungen beachten: Orthopädische Probleme wie Arthrosen oder Hallux valgus können die Gangunsicherheit einer PNP vortäuschen, ebenso wie die altersbedingte Verschlechterung von Gleichgewicht, Sehen, Hören und zentraler Regulation der posturalen Kontrolle.

## Elektrophysiologische Untersuchungen

Diese werden zur pathophysiologischen Einordnung von PNP, aber auch zu deren Objektivierung eingesetzt. Die Unterscheidung in axonal oder demyelinisierend ist wesentlicher Bestandteil der Befundung. Bei axonalen Polyneuropathien liegt ein primär axonaler Nervenschaden mit einer Amplitudenminderung der motorischen oder sensiblen Antwortpotenziale vor. Bei einer demyelinisierenden Polyneuropathie findet sich eine Verlängerung der Nervenleitgeschwindigkeit (NLG), da durch einen primären Schaden der Myelinscheide die Nervenleitung verlangsamt ist. Zu den axonalen PNP zählen beispielsweise die diabetische PNP, die chronische idiopathische axonale Polyneuropathie (CIAP), die PNP bei Vitaminmangel, aber auch die meisten toxischen Polyneuropathien. Zu den – wesentlich selteneren – demyelinisierenden PNP zählen einige hereditär-motorische sensible Neuropathien (HMSN), die PNP bei Gammopathien, aber auch die CIDP (chronisch inflammatorische Polyneuropathie).

Die meisten Parameter der Elektroneurographie verändern sich mit dem Alter; so nehmen die Amplituden der sensiblen, aber auch der motorischen Potenziale ab [8].

## Häufige PNP des höheren Lebensalters

### Chronische idiopathische axonale Polyneuropathie (CIAP)/kryptogene Neuropathie

Die CIAP ist die häufigste Neuropathie im Alter [5]. Klinisch präsentiert sich ein mildes und sehr langsam progredientes Bild in Form distaler Sensibilitätsstörungen an den Beinen, einer geringen sensiblen Ataxie und neuropathischen Schmerzen [9]. Elektrophysiologisch handelt es sich um eine axonale Neuropathie. Differenzialdiagnostisch müssen metabolische, toxische und systemische Ursachen ausgeschlossen werden. Die CIAP kann mit einem metabolischen Syndrom assoziiert sein, was einen präventiv-therapeutischen Ansatz ermöglicht [10].

### Diabetische Polyneuropathie (DPNP)

Jenseits des 60. Lebensjahres treten DPNP bei > 50 % der Patient:innen mit Diabetes auf. Am häufigsten ist die *distal symmetrische*, sensibel betonte und zusätzlich autonome Neuropathie. Dabei kommt es distal betont zu strumpfförmigen Parästhesien, häufig finden sich Pallhypästhesie (reduzierte Wahrnehmung von Vibration, Berührung und Druck) und Hypalgesie (reduzierte Schmerzwahrnehmung). Im Verlauf kann es zu motorischen Ausfällen, z. B. zum Verlust der Muskeleigenreflexe, und zu schlaffen Lähmungen kommen. Autonome Symptome umfassen orthostatische Dysregulation, Obstipation, Diarrhoe sowie Anhidrose. Die *diabetische Ophthalmoplegie* mit Ausfällen der Hirnnerven III, IV und VI setzt oft mit heftigen orbitalen Schmerzen ein, gefolgt von Doppelbildern. Häufig remittieren die Symptome innerhalb von 4–6 Wochen. Die *diabetische Radikulopathie* ist durch plötzlich auftretende Schmerzen charakterisiert, die gürtelförmig ein- oder beidseitig von der Wirbelsäule zur Brust- oder Bauchregion ziehen.

### Lumbosakrale Radikulo-Plexo-Neuropathie (LRPN)

Die *LRPN* tritt im medianen Alter von 65–70 Jahren auf [11, 12]. Akut einsetzende heftige Schmerzen ziehen von lumbal zur Ventralseite eines Oberschenkels, innerhalb von Wochen bis Monaten entwickeln sich Lähmungen und Atrophien der Beckengürtel- und Oberschenkelmuskulatur, potenziell begleitet von einem deutlichen Gewichtsverlust. Sensibilitätsstörungen stehen im Hintergrund. Die Symptomatik beginnt meist einseitig, breitet sich auf die andere Seite aus, bleibt aber häufig asymmetrisch. Die Erkrankung verläuft monophasisch, eine langsame Besserung beginnt oft ab dem 6. Monat. Die Erkrankung kann idiopathisch oder als diabetische LRPN (diabetische Amyotrophie) auftreten. Die Differenzialdiagnose zu radikulären Ausfällen kann bildgebende Verfahren notwendig machen. Da die Histologie von Nervenbiopsaten eine epineurale Vaskulitis zeigte, sind intravenöse Steroide oder Immunglobuline als Therapieversuche vertretbar [11].

### Polyneuropathien bei monoklonalen Gammopathien unspezifischer Signifikanz (MGUS)

Im Alter finden sich vermehrt Paraproteinämien (monoklonale Gammopathien). Diese können im Kontext hämatologischer Erkrankungen (Multiples Myelom, Morbus Waldenström) auftreten, meist findet sich aber eine MGUS: Diese Veränderung tritt bei 5,3 % der > 70-Jährigen und bei 10 % der > 80-Jährigen auf und ist bei 10 % der Patienten mit kryptogener Neuropathie nachweisbar [13]. Die Symptomatik variiert, häufig findet sich eine langsam progrediente distal betonte sensible Neuropathie. Im Verlauf können motorische Defizite sowie selten autonome Symptome (orthostatische Dysregulation) auftreten.

### Polyneuropathien durch Vitaminmangel

Eine Mangelernährung als Ursache von Hypovitaminosen findet sich bei gestörter enteraler Resorption, meistens betrifft der Mangel B‑Vitamine. Sowohl ein Mangel an B_1_ (Thiamin), B_6_ (Pyridoxin) wie auch B_12_ (Cobalamin) kann zu Neuropathien führen. Am häufigsten ist der B_12_-Mangel, der sich bei bis zu 6 % der < 60-Jährigen und bis zu 20 % bei > 60-Jährigen findet [14]. Typisches klinisches Bild ist die Kombination von Rückenmarkssymptomen mit denen einer Polyneuropathie [15]. Ein klinischer Hinweis auf einen B_12_-Mangel ist das gleichzeitige Auftreten von sensiblen Symptomen an Händen und Füßen.

Die isolierte Messung von B_12_ reicht nicht aus, die ergänzende Bestimmung von Homocystein, Methylmalonat (MMS) und Holo-Transcobalamin (Holo-TC) erhöht die diagnostische Sensitivität. Bei Niereninsuffizienz sind MMS und Holo-TC allerdings nur eingeschränkt verwertbar [15].

Bei B_12_-Mangel soll eine sofortige parenterale Substitution erfolgen, da eine Besserung nur bei frühem Therapiebeginn zu erwarten ist. Rezente Beobachtungen legen eine Assoziation von L‑Dopa-Therapie und B_12_-Mangel nahe, daher sollte bei L‑Dopa-Langzeit-Therapie und PNP an einen B_12_-Mangel gedacht werden [16].

### Paraneoplastische Neuropathien

Zwei Drittel aller malignen Erkrankungen treten jenseits des 65. Lebensjahres auf, das PNS kann dabei in unterschiedlicher Form involviert sein. Eine lokale Tumorinfiltration kann Hirnnerven, Nervenwurzeln, Plexus brachialis- und lumbosacralis oder periphere Nerven treffen. Antineoplastische Therapeutika können Nebenwirkungen entfalten, die periphere Nerven schädigen [15]. Eine Neuropathie als paraneoplastisches Syndrom kann der Entdeckung der Tumorerkrankung vorausgehen. Häufigste Ursache ist das kleinzellige Bronchialkarzinom, gefolgt von Mamma- und Ovarialkarzinomen sowie Lymphomen. Die typische Präsentation ist die einer subakuten sensiblen Neuronopathie mit multifokalem und asymmetrischem Beginn. Früh sind die Arme und häufig auch Gesicht und Rumpf betroffen, neuropathische Schmerzen sind häufig, ebenso eine ausgeprägte sensible Extremitätenataxie. Bei der Mehrzahl der Patienten können im Serum antineuronale Antikörper nachgewiesen werden [17].

## Therapeutische Herausforderungen im Alter

Neben der Behandlung der Grunderkrankungen ist im Alter die Behandlung des *neuropathischen Schmerzes *oft herausfordernd. Studiendaten für alte Patient:innen stehen auch hier kaum zur Verfügung. Alte Menschen sind hinsichtlich unerwünschter Arzneimittelwirkungen aufgrund von Multimorbidität, Mangelernährung, Organinsuffizienzen, Multimedikation, Sarkopenie und Frailty deutlich vulnerabler als junge [18]. Eine Zusammenfassung medikamentöser Therapieoptionen bietet Tab. 2. Eine eingeschränkte Nierenfunktion spielt eine besondere Rolle bei der Therapie mit Gabapentin und Pregabalin, da ein Anpassen der maximalen Tagesdosis zu beachten ist. Bei der Therapie mit SNRI (Duloxetin, Venlafaxin) und SSRI (Citalopram, Paroxetin) ist das Risiko einer Hyponatriämie zu erwähnen, bei Multimedikation ist die QT-Zeit zu kontrollieren. Die Gabe von Antidepressiva ist im Alter mit einem erhöhten Sturzrisiko verbunden [19]. Bei Opiattherapie ist ein erhöhtes Nebenwirkungsrisiko seitens des ZNS gegeben. Verschlechterungen kognitiver Funktionen bis hin zum Delir sind möglich. Die topische Anwendung von Capsaicin ist häufig durch die Fragilität der Haut limitiert.

**Tab. 2:** Medikamentöse Therapie neuropathischer Schmerzen (Auswahl)

Wirkstoff	Startdosis	Übliche wirksame Dosis	Wichtige Hinweise
Gabapentin	100 mg abends, dann langsam steigern	900–2400 mg/Tag, max. 3600 mg/Tag	Dosisanpassung bei Niereninsuffizienz; oft Schwindel, Müdigkeit
Pregabalin	25–50 mg abends oder 2 × 50 mg/Tag	150–300 mg/Tag, max. 600 mg/Tag	Dosisanpassung bei Niereninsuffizienz; häufig Schwindel, Müdigkeit, Ödeme
Duloxetin	30 mg morgens	60 mg/Tag	Bei älteren Patient:innen besseres Nebenwirkungsprofil als trizyklische Antidepressiva; auf Blutdruck und Leberfunktion achten
Tramadol	25–50 mg, vorsichtig titrieren, in retardierter Form bessere Verträglichkeit	Nur kurzfristig, möglichst niedrig dosiert	Nicht erste Wahl; im Alter wegen des Risikos für Stürze, Übelkeit und Delir zurückhaltend einsetzen
Oxycodon	10–20 mg in retardierter Form	20–80 mg	Nicht erste Wahl; Risiko für Delir und Sedierung. Übelkeit und Obstipation häufig. Ab 30 mg Tagesdosis erhöhtes Suchtrisiko
Morphin	2 × 10 mg in retardierter Form	40–120 mg	Risiko für Delir und Sedierung. Übelkeit und Obstipation häufig

Trizyklische Antidepressiva (Amitriptylin, Imipramin) sind aufgrund des anticholinergen Nebenwirkungsprofils nicht angeführt. Die Wirksamkeit weiterer Antidepressiva (u. a. Venlafaxin, Citalopram, Paroxetin) ist weniger gut belegt. Andere Opioide (u. a. Hydromorphon, Fentanyl, Buprenorphin) sind aufgrund fehlender Daten nicht angeführt

Neuropathische Schmerzen werden auch durch nichtmedikamentöse Behandlungen wie Wärme- und Kälteanwendungen sowie Elektrotherapie positiv beeinflusst. Maßnahmen zur Kräftigung der (unteren) Extremitäten und zur Reduktion von Sturzbedenken, sensomotorisches Training zur Verbesserung der Gleichgewichtsfähigkeit, Aufstehtraining, Gangschulung und das Anpassen von Gehhilfen ergänzen das Spektrum. Bei Neuropathien mit Prädisposition zu trophischen Störungen (z. B. diabetische Neuropathie) sind Maßnahmen zur Prävention von Ulzera erforderlich.

Bei alten Menschen ist auch die Abklärung der funktionellen Fähigkeiten essenziell, hier kommt das geriatrische Assessment zum Zug. Besonderes Augenmerk ist auf das Einschätzen von Mobilität und Sturzrisiko zu legen. In Hinblick auf das Frakturrisiko ist die Abklärung einer möglichen Osteoporose erforderlich.

## Funktionelle Auswirkungen und Auswirkungen auf die Kommunikationsfähigkeit

Neben den neurologischen Symptomen zeigen PNP substanzielle Auswirkungen auf psychosoziale Dimensionen und gesellschaftliche Teilhabe. Nach dem WHO-Rahmenkonzept der International Classification of Functioning, Disability and Health (ICF) ist eine PNP als Erkrankung mit potenziellen Aktivitäts- und Partizipationseinschränkungen zu sehen, die in Wechselwirkung mit Kontextfaktoren stehen. Die Kommunikationsfähigkeit Betroffener mit ihrer Umwelt wird dabei vielfältig beeinträchtigt: So führen sensorische Defizite (Hypästhesie, Dysästhesie), motorische Einschränkungen (Gangunsicherheit, Muskelschwäche) und neuropathischer Schmerz zu Aktivitätslimitationen (z. B. eingeschränkte Mobilität, reduzierte Feinmotorik). Diese können die soziale Partizipation beeinträchtigen, etwa bei Freizeitaktivitäten oder in familiären Rollen. Studien belegen konsistent eine signifikant reduzierte gesundheitsbezogene Lebensqualität (HRQoL) bei Patient:innen mit PNP im Vergleich zu Kontrollgruppen. Lin et al. zeigten signifikant niedrigere Werte in den physischen und psychologischen Domänen, auch die soziale Dimension war betroffen. Die Beeinträchtigung korrelierte mit Parametern der Small-Fiber-Dysfunktion, was auf einen direkten Zusammenhang zwischen neurologischer Schädigung und subjektiver Lebensqualität hinweist [20]. Eine multizentrische Untersuchung zeigte für die soziale Funktionsfähigkeit eine signifikante Einbuße bei höherem Behinderungsgrad. Damit wird deutlich, dass neurologische Defizite auch interpersonelle und gesellschaftliche Interaktionen negativ beeinflussen [21]. Übersichtsarbeiten zu schmerzhaften PNP bestätigen diese Befunde: Neuropathischer Schmerz ist mit einer Reduktion sozialer Aktivitäten, eingeschränkter Arbeitsfähigkeit und emotionaler Belastung assoziiert. Die soziale Beeinträchtigung ist dabei nicht allein Folge motorischer Defizite, sondern wird wesentlich durch Schmerzintensität, Schlafstörungen und Fatigue vermittelt [22]. Neuropathischer Schmerz stellt eine der wichtigsten Determinanten psychosozialer Beeinträchtigung dar. Die Schmerzintensität korreliert signifikant mit Einschränkungen in Alltagsaktivitäten und sozialer Funktionsfähigkeit [23]. Stärkere Schmerzen gehen mit reduzierter Mobilität, verminderter Teilnahme an Freizeitaktivitäten und eingeschränkter Leistungsfähigkeit einher. Der Mechanismus ist multifaktoriell: Chronischer Schmerz begünstigt Schlafstörungen, Fatigue und affektive Symptome (Depression, Angst), die wiederum soziale Interaktion und Teilhabe negativ beeinflussen. Schmerz ist damit eine vermittelnde Variable zwischen neurologischer Schädigung und sozialer Isolation. Auch psychosoziale Kontextfaktoren spielen eine wesentliche Rolle. Wahrgenommene soziale Unterstützung ist ein unabhängiger Prädiktor für die mentale Lebensqualität – auch nach Adjustierung für Schmerzintensität, Alter, Geschlecht und Erkrankungsschwere. Patient:innen mit besserer sozialer Unterstützung wiesen signifikant bessere Werte in den mentalen Dimensionen der Lebensqualität auf [24]. Diese Ergebnisse unterstreichen die Relevanz sozialer Ressourcen: Soziale Unterstützung kann negative Auswirkungen körperlicher Einschränkungen mildern und adaptive Coping-Strategien fördern.

## Fazit für die Praxis

- Die vorliegenden Befunde belegen, dass das Krankheitsbild PNP weit über die periphere Nervenschädigung hinausgeht und als Entität mit substanziellen sozialen Konsequenzen zu verstehen ist.
- Das PNS ist innerhalb des Nervensystems für die komplexe Signalverarbeitung zwischen ZNS und Umgebung verantwortlich. Sensorische Nerven leiten Informationen zum ZNS, motorische Nerven leiten Signale des ZNS zu den Muskeln, das vegetative (autonome) Nervensystem versorgt die inneren Organe. Erkrankungen des PNS (PNP) beeinträchtigen somit die Kommunikation innerhalb des Nervensystems mit weitreichenden Folgen für die Interaktion des Individuums mit seinen Umwelten.
- Die Beeinträchtigung von Kommunikation und sozialer Teilhabe ergibt sich aus dem Zusammenspiel von neurologischer Funktionsstörung, neuropathischem Schmerz und psychosozialen Kontextfaktoren. Insbesondere neuropathischer Schmerz fungiert als zentraler Mediator zwischen körperlicher Einschränkung und sozialer Isolation.
- Studien zeigen, dass soziale Unterstützung eine protektive Rolle einnimmt und die mentale Lebensqualität signifikant beeinflusst. Im Sinne des bio-psycho-sozialen Modells sollte die Behandlung von Polyneuropathien daher multidimensional erfolgen und neben pharmakologischer Schmerztherapie auch rehabilitative, psychosoziale und sozialmedizinische Interventionen umfassen.
- Durch eine integrativ-geriatrische Perspektive lässt sich der Verlust sozialer Teilhabe nachhaltig reduzieren.

## Data Availability

Alle dieser Arbeit zugrunde liegenden Daten sind in diesem Artikel enthalten.
